# Whole Exome Sequencing Identifies Two Novel Mutations in a Patient with UC Associated with PSC and SSA

**DOI:** 10.1155/2021/9936932

**Published:** 2021-09-10

**Authors:** Dong Wu, Dan Chen, Wen Shi, Wei Liu, Weixun Zhou, Jiaming Qian

**Affiliations:** ^1^Department of Gastroenterology, Peking Union Medical College Hospital, Chinese Academy of Medical Sciences & Peking Union Medical College Hospital, Beijing 100730, China; ^2^Center for Rare Diseases Research, Peking Union Medical College Hospital, Chinese Academy of Medical Sciences & Peking Union Medical College Hospital, Beijing 100730, China; ^3^Department of Radiology, Peking Union Medical College Hospital, Chinese Academy of Medical Sciences & Peking Union Medical College Hospital, Beijing 100730, China; ^4^Department of Pathology, Peking Union Medical College Hospital, Chinese Academy of Medical Sciences & Peking Union Medical College Hospital, Beijing 100730, China

## Abstract

**Background:**

Patients diagnosed with ulcerative colitis (UC) associated with primary sclerosis cholangitis (PSC) and sessile serrated adenoma (SSA) are rare. The present study aimed to identify the potential causative gene mutation in a patient with UC associated with PSC and SSA.

**Methods:**

DNA was extracted from the blood sample and tissue sample of SSA, followed by the whole exome sequencing (WES) analysis. Bioinformatics analysis was utilized to predict the deleteriousness of the identified variants. Multiple sequence alignment and conserved protein domain analyses were performed using online software. Sanger sequencing was used to validate the identified variants. Expression and diagnostic analysis of identified mutated genes was performed in the GSE119600 dataset (peripheral blood samples of *PSC* and *UC*) and GSE43841 dataset (tumor samples of *SSA*).

**Results:**

In the present study, a total of 842 single nucleotide variants (SNVs) in 728 genes were identified in the blood sample. Two variants, integrin beta 4 (*ITGB4*) (c.C2503G; p.P835A) and a mucin 3A (*MUC3A*) (c.C1019T; p.P340L), were further analyzed. *MUC3A* was associated with inflammatory bowel disease. Sanger sequence in blood revealed that the *ITGB4* mutation was fully cosegregated with the result of WES in the patient. Additionally, a variant, tumor protein p53 gene (*TP53*) (c.86delA; p.N29Tfs^*∗*^15) was identified in the tissue sample of SSA. Compared to that in normal controls, *ITGB4* was upregulated in both UC and PSC, *MUC3A* was, respectively, upregulated and downregulated in PSC and UC, and *TP53* was downregulated in SSA. *ITGB4* and *TP53* had a potential diagnostic value for UC, PSC and SSA.

**Conclusions:**

The present study demonstrated that the *ITGB4* (c.C2503G; p.P835A) and *MUC3A* (c.C1019T; p.P340L) mutations may be the potential causative variants in a patient with UC associated with PSC and SSA. *TP53* (c.86delA; p.N29Tfs^*∗*^15) mutation may be associated with SSA in this patient.

## 1. Background

Primary sclerosis cholangitis (PSC) is a chronic disease with inflammation and fibrotic obliteration of the intra and extrahepatic bile duct, which can lead to bile stasis and hepatic fibrosis [1, 2]. PSC is associated with underlying inflammatory bowel disease (IBD), mainly ulcerative colitis (UC) [1]. There is no effective medical treatment for this condition, and liver transplantation represents the treatment of choice for patients with end-stage [3]. In Western countries, approximately 52–90% of patients with PSC have concurrent UC [4–8]. Meanwhile, the number of patients with UC, as well as with other types of IBDs, continues to increase in Western countries [9, 10]. Several current reports suggest that patients with PSC associated with UC (PSC-UC) have increased gradually in some European populations [11]. The involvement of gut bacteria in PSC-UC patients is widely accepted [12, 13]. Accumulating evidence suggested that gut dysbiosis and abnormal bile acid metabolites are involved in PSC [14]. However, the pathogenetic mechanism of PSC is far from clear. Furthermore, patients with PSC-IBD have an increased risk of colon cancer and cholangiocarcinoma compared with those with IBD alone [15].

Sessile serrated adenomas (SSA) give rise to 20–30% of colorectal cancer (CRC). SSA displays endoscopic features which present challenges in diagnosis and surveillance. SSA are increasingly found in colectomy specimens from CRC patients. SSA differs from conventional adenomas in several respects. They arise at any age and are over represented in young patients [5, 6]. Histologically, they vary little in appearance for the majority of their dwell time prior to the development of dysplasia. However, once dysplasia develops, SSA rapidly progresses to CRC [7]. SSAs are thought to be responsible for “interval” CRCs which arise within the colonoscopy surveillance interval [7]. Genetic factors that contribute to development of SSA are not well understood, but microsatellite instability (MSI) is thought to be involved. Rajagopalan et al. [16] used the HumanMethylation 450 array to identify methylated genes in SSA compared with normal mucosa, followed by RNA sequencing. Three genes were methylated and downregulated in SSA, including bone morphogenetic protein 3 (*BMP3*), erythrocyte membrane protein band 4.1 like 3 (*EPB41L3*), and cystathionine beta-synthase (*CBS*). Using a customised methylation microarray, Beach et al. [17] identified 32 genes which were methylated in SSAs compared with normal mucosa and performed immunohistochemistry for the six most promising candidates.

Whole exome sequencing (WES) is widely used to explore the genetic mechanism of rare diseases [18–20]. The present study investigated a patient with UC associated with PSC and SSA. The results on the inheritance of mutant genes may provide novel information regarding the pathological mechanism underlying this unusual condition.

## 2. Methods

### 2.1. Patients

For the purpose of this study, a patient with UC associated with PSC and SSA was recruited. The diagnosis of UC was made due to clinical symptoms, endoscopic examination, and biopsy of the colon mucosa. The diagnosis of PSC was based on radiological imaging and liver biopsy. The diagnosis of SSA was based on endoscopic features and histological investigation. The patient's peripheral blood was collected. Genomic DNA was extracted for WES. The tissue sample of SSA from a colonoscopy biopsy was also used for WES analysis. Our study was approved by the Research Ethics Committee of the Peking Union Medical College Hospital (approval number 2603, 2898). The written informed consent was obtained from this patient before the study.

### 2.2. Analysis of Exome Capture

The genomic DNA was extracted from the blood sample according to the standard procedures. The 3 *μ*g of genomic DNA was fragmented with approximately 200 bp, ligated with adapters, and amplified by ligation-mediated polymerase chain reaction (PCR). The qualified genomic DNA was used for exome capture and high-throughput sequencing. SureSelect XT Library Prep Kit was used to perform exome target enrichment. The captured library was sequenced on the Illumina HiSeq X-Ten sequencer with paired-end 125 bp and mean coverage of 200x [21].

### 2.3. Analysis of Basic Biological Information

The FastQC and SeqPrep were utilized to detect the quality of primitive data of exome sequencing. Raw data were filtered by removing the adapter, contaminating reads and low-quality reads via SeqPrep and sickle, and the remains were the clean ones. The exome sequencing clean reads were mapped to the reference human genome sequence (hg19) (http://hgdownload.soe.ucsc.edu/goldenPath/hg19/bigZips/) using the Burrows–Wheeler Alignment (BWA) tool (http://bio-bwa.sourceforge.net/bwa.shtml), which can do short reads alignment to a reference genome and support paired-end mapping. The sequence alignment/map (SAM) file was then generated. Picard tool (http://picard.sourceforge.net/) was used to mark and exclude the duplicate reads. Variants (single nucleotide variants (SNVs), insertions, and deletions) calling was performed using the Genome Analysis Toolkit (GATK) [22].

### 2.4. Functional Annotation of Hotspot Mutation Genes

In order to investigate the biological function of hotspot mutation genes, 73 hotspot mutation genes were analyzed in the Gene Ontology (GO) functional categories, the Online Mendelian Inheritance in Man (OMIM), and the Kyoto Encyclopedia of Genes and Genomes (KEGG) biochemical pathway using the GeneCodis3 (http://genecodis.cnb.csic.es/analysis).

### 2.5. Sanger Sequencing for Variant Validation

After the systematic filtering, two candidate variants *ITGB4* (c.C2503G; p.P835A) and *MUC3A* (c.C1019T; p.P340L) were selected for variant validation. Genomic DNA was prepared from the patient. After oligonucleotide primer was designed by using Primer-Premier 5.0 (Premier Biosoft International, Palo Alto, CA, USA). PCR analyses and Sanger sequencing were then performed. The amplification process was 15 min at 95°C followed by 40 cycles of 10 sec at 95°C, 30 sec at 55°C, 32 sec at 72°C, and 15 sec at 95°C, 60 sec at 60°C, and 15 sec extension at 95°C. The PCR products were used for Sanger sequencing.

### 2.6. In Silico Analysis

The multiple sequence alignment of *ITGB4* and *TAS2R46* in different species was performed using an online tool (https://www.ncbi.nlm.nih.gov/tools/cobalt/cobalt.cgi?LINK_LOC=BlastHomeLink). In addition, Conserved Domain Search Service (http://www.ncbi.nlm.nih.gov/Structure/cdd/wrpsb.cgi) was used to identify the conserved protein domains.

### 2.7. Expression and Diagnostic Analysis of Mutated Genes

The GSE119600 dataset (peripheral blood samples, involving 45 PSC cases, 93 UC cases, and 47 normal controls) and GSE43841 dataset (tumor samples, involving 6 SSA cases and 6 normal controls) were used for expression and diagnostic analysis of mutated genes. The ROC analysis was used to assess the diagnostic value of mutated genes. The expression result of mutated genes was shown by box plots.

## 3. Results

### 3.1. Clinical Manifestations

**Figure 1:**
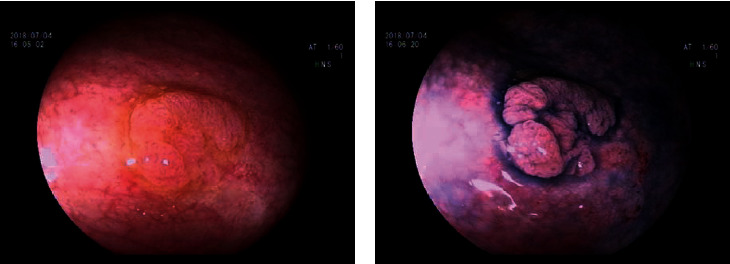
Flat polyp with subtle margin at the cecum ((a) white light; (b) indigo carmine).

**Figure 2:**
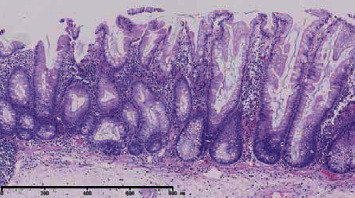
Serrated hyperplasia of the crypts and basal dilation of some crypts, consistent with SSA.

**Figure 3:**
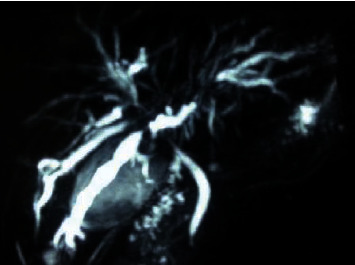
Multiple stricture and dilation of intrahepatic bile ducts.

**Figure 4:**
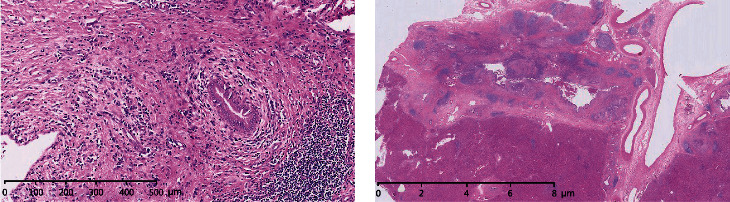
(a) Liver biopsy revealing portal infiltration of lymphocytes, plasmacytes, and scattered eosinophils with fibrosis and pseudolobule (H&E, 10 × 10). (b) Histological examination demonstrating neutrophilic microabscesses in the crypts (H&E, 10 × 20).

At present study, one male patient (63 years old) who was diagnosed with UC associated with PSC and SSA was included in the study. He had bloody diarrhea and lower abdominal pain for 8 years before admission. On colonoscopic examination, diffuse edema, erosion, and petechial hemorrhage were noted along the whole colon and rectum. The diagnosis of UC was made based on clinical, endoscopic, and histological data. A flat polyp with a size of 0.6 cm was detected in the cecum and was resected (Figure 1). Histological diagnosis of SSA was made according to serrated hyperplasia and basal dilation of crypts (Figure 2). PSC was diagnosed based on radiological image (Figure 3) and liver biopsy (Figure 4(a)). Histological examination demonstrated neutrophilic microabscesses in the crypts and infiltration with mononuclear cells and plasma cells in the lamina propria (Figure 4(b)). Figure 4(a) discloses onion skin-like changes, lymphocyte infiltration plasmacytes, and scattered eosinophils with fibrosis and pseudolobulation. Two portal areas had hyperplastic ductule.

### 3.2. Genetic and In Silico Analysis

Genomic DNA samples in the blood sample from the patient were analyzed. A total of 842 single nucleotide variants (SNVs) in 728 genes were identified. Among which, 26 genes had three or more rare missense mutations, and 47 genes had two rare missense mutations. Based on the WES in the patient and subsequent Sanger sequencing results, 6 SNVs, the c.C2503G mutation (a substitution from P to A) in *ITGB4* (Chr17: 73736495), the c.C1019T mutation (a substitution from P to L) in *MUC3A* (Chr7: 100550438), the c.G418C mutation (a substitution from D to H) in *HLA-A*, the c.C40664T mutation (a substitution from P to L) in *MUC16*, the c.T869A mutation (a substitution from F to Y) in *TAS2R46*, and c.C3414A mutation (a substitution from D to E) in *MUC5B* were likely to be associated with UC associated with PSC and SSA. In the tissue sample of the patient when diagnosed with SSA, *TP53* mutation (c.86delA; p.N29Tfs^*∗*^15) (Chr17: 7579710) was identified. Interestingly, the missense mutation of *ITGB4*, *MUC16*, and TP53 is found in the tumor tissue of CRC in the Cancer Genome Atlas (TCGA) dataset (involving 471 CRC cases and 41 normal controls). It is suggested that mutations of *ITGB4*, *MUC16*, and TP53 are significantly associated with both SSA and CRC (Supplementary Figure 1).

### 3.3. Functional Annotation of 73 Hotspot Mutation Genes

In Supplementary Figure 2, based on the GO, KEGG, and OMIM enrichment analyses, *TAS2R31*, *TAS2R46*, and *TAS2R43* were significantly enriched in taste transduction (FDR = 0.005854). *MUC3A* was associated with inflammatory bowel disease 11 (FDR = 0.004677). *MUC5B* was associated with pulmonary fibrosis idiopathic (FDR = 0.013961). *HLA-A* was associated with severe cutaneous adverse reaction and (FDR = 0.028276) spondyloarthropathy (FDR = 0.028276). *ITGB4* was involved in epidermolysis bullosa simplex (FDR = 0.008169).

### 3.4. In Silico Analysis

**Figure 5:**
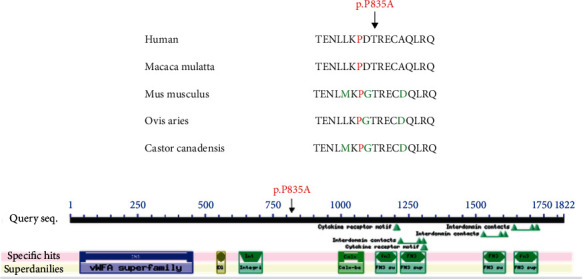
(a) Affected amino acid residue of *ITGB4* highly conserved between different species. (b) Conserved domains in fatty acid synthase.

**Figure 6:**
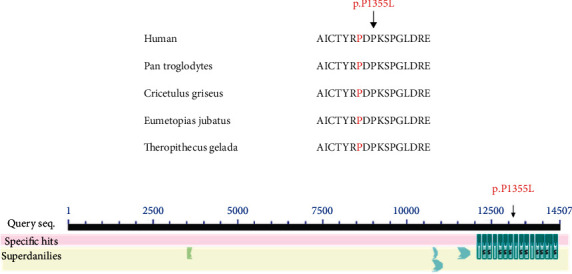
(a) Affected amino acid residue of *MUC16* highly conserved between different species. (b) Conserved domains in fatty acid synthase.

The c.C2503G mutation in *ITGB4* resulted in a protein alteration of p.P835A. In addition, the c.C40664T mutation in *MUC16* resulted in a protein alteration of p.P13555L. Furthermore, it was highly conserved across several species, including humans, *Macaca mulatta*, *Mus musculus*, *Ovis aries*, and *Castor canadensis* (Figure 5). Furthermore, the amino acid at position 13555 in the *MUC16* protein sequence was located in the SEA domain, and it was highly conserved across several species, including humans, *Pan troglodytes*, *Cricetulus griseus*, *Eumetopias jubatus*, and *Theropithecus gelada* (Figure 6).

### 3.5. Sanger Sequencing of the Candidate Causative Variants

**Figure 7:**
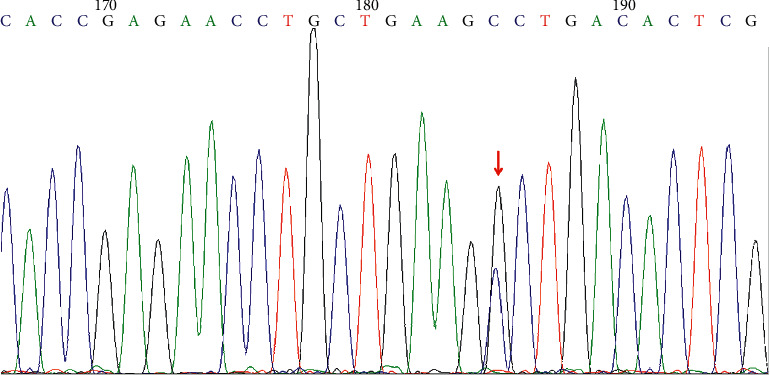
Sanger validation results of *ITGB4* variant in the patient with UC associated with PSC and SSA. Arrow represents the mutation site.

In order to further confirm the c.C2503G mutation in UC associated with PSC and SSA, Sanger sequencing was performed on the patient. The results demonstrated that the c.C2503G mutation in *ITGB4* was fully cosegregated with patient recruited for WES (Figure 7).

### 3.6. Expression and Diagnostic Analysis of Mutated Genes

**Figure 8:**
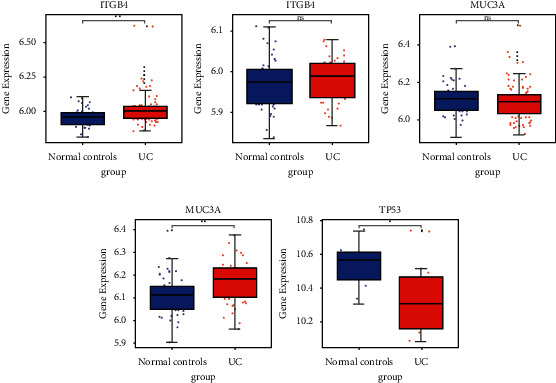
Expression box plots of *ITGB4*, *MUC3A*, and *TP53* in peripheral blood of UC and PSC and *TP53* in tumor of SSA in the GSE43841 dataset.

**Figure 9:**
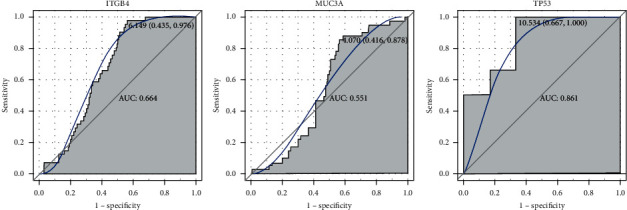
The ROC curves of *ITGB4* and *MUC3A* in UC and PSC and *TP53* in SSA.

First, we investigated the expression of *ITGB4* and *MUC3A* in peripheral blood of UC and PSC in the GSE119600 dataset and the expression of *TP53* in tumor of SSA the GSE43841 dataset (Figure 8). The result showed that *ITGB4* was upregulated in both UC and PSC, compared to that in normal controls. *MUC3A* was upregulated in PSC and downregulated in UC, compared to that in normal controls. *TP53* was downregulated in SSA, compared to that in normal controls. It is suggested that mutation of above genes may be associated with gene expression. Second, we performed the ROC analysis to evaluate the diagnostic value of *ITGB4*, *MUC3A*, and *TP53* (Figure 9). The ROC result showed that the AUC of *ITGB4* and *TP53* was more than 0.6, which suggested that they had a diagnostic value for UC, PSC, and SSA.

## 4. Discussion

Patients who were diagnosed with UC associated with PSC and SSA were clinically rare. UC is currently affecting more than 3.5 million people in Europe and North America [23]. The disease is recrudescent, and the main symptoms during a relapse are diarrhea, rectal bleeding, and abdominal pain [24]. Approximately every 20 patients with IBD are affected by PSC, a chronic bile duct disease characterized by obliterative fibrosis. This is an incurable, progressive condition associated with an increased risk of cholangiocarcinoma and colon cancer [25, 26]. At present, genetic mechanisms underlying UC associated with PSC pathogenesis remain to be fully elucidated. As previously reported, David Ellinghaus identified two novel risk locis, G protein-coupled receptor 35 (*GPR35*) and transcription factor 4 (*TCF4*), at genome-wide significance levels. GPR35 shows associations in both UC and PSC, whereas *TCF4* represents a PSC risk locus not associated with UC [27]. WES technology is an effective method for identifying potential causative genes in disease phenotypes [28]. In the present study, WES was performed to identify potential causative genes in the patient who was diagnosed with UC associated with PSC and SSA. Two mutations (c.C2503G and c.C1019T) in *ITGB4* and *MUC3A* genes, respectively, were revealed to be associated with UC associated with PSC and SSA. *MUC3A* was associated with inflammatory bowel disease. In addition, Sanger sequencing validated *ITGB4* mutation in the patient.

Our result demonstrated the crucial role of *MUC3A* in the development of UC associated with PSC and SSA. Mucins are classified into 11 membrane-bound mucins and 7 secreted mucins. Analogous to MUC3A, MUC1, MUC3B, MUC4, MUC12, MUC13, MUC15, MUC16, MUC17, MUC20, and MUC21 are all membrane-bound mucins [29]. Membrane-bound mucins share conserved domains, such as sea urchin sperm protein enterokinase and agrin (SEA) domains or epidermal growth factor-like (EGF). Due to their localization and structure at the cell surface, they may participate in cell signaling, cell-matrix, and cell-cell interactions, modulating biological properties under normal and pathological conditions [30]. *MUC3A* was located in chromosome 7q22 with tandem repeats of 17 amino acids and categorized as a membrane-associated mucin [31]. *MUC3A* includes two extracellular cysteine-rich epidermal growth factor-like domains that maybe implicated in cell proliferation through growth factors [31]. Poor prognosis with abnormal high *MUC3A* expression has been researched in breast, pancreatic, gastric, colorectal, appendiceal, and prostate cancer [32–37]. Zimmer et al. revealed an aberrant expression of *MUC2* and *MUC3* of the gastric epithelium in Menetrier's disease during remission of UC [38]. Previous study reported that B-Raf protooncogene, serine/threonine kinase (*BRAF*), and KRAS protooncogene, GTPase (*KRAS*) mutations, are early molecular alterations in serrated lesions and are mutually exclusive in colorectal neoplasms [16, 17]. In the present study, a mutation (c.C1019T; p.P340L) in the *MUC3A* gene was detected in the patient with UC associated with PSC and SSA. Moreover, *MUC3A* was, respectively, upregulated and downregulated in the peripheral blood of PSC and UC patients. It is suggested that mutation of *MUC3A* may be associated with its expression. Interestingly, *ITGB4* had a potential diagnostic value for UC and PSC. In addition, *MUC3A* was highly conserved among different species, including humans, *Ovis aries*, *Pteropus alecto*, *Theropithecus gelada*, and *Octodon degus*. *MUC3A* was enriched in the maintenance of gastrointestinal epithelium and extracellular matrix structural constituent and associated with inflammatory bowel disease. Therefore, this *MUC3A* mutation may be considered one candidate molecule in the pathogenesis of UC associated with PSC and SSA.

Microsatellites are short tandem repeats that are found throughout the human genome [39]. Compared with normal tissue, the microsatellite of tumor tissue changes the length of the microsatellite due to the insertion or deletion of repeating units, called microsatellite instability (MSI) [40]. Numerous studies have shown that MSI is caused by defects in the mismatch repair (MMR) gene and is closely related to tumorigenesis [41, 42]. MSI has been clinically used as an important molecular marker for the prognosis of CRC and the development of adjuvant therapy and is used to assist in the screening of Lynch syndrome [43]. SSA shares molecular features with colon tumors that have MSI and a methylated phenotype, indicating that these lesions are precursors that progress via the serrated neoplasia pathway. But the genetic mutation in the pathogenesis of SSA is needed to be fully clarified. As a member of the integrin family, *ITGB4* generally forms heterodimers with integrin 6 to achieve its biological functions, and integrin molecules can also coordinate with receptor tyrosine kinases (RTKs) to regulate downstream signaling pathways. Recent studies demonstrated that *ITGB4* played an important role in promoting tumorigenesis in prostate cancer [44], breast cancer [45], gastric cancer [46], and lung squamous cell carcinoma [47]. In the present study, a mutation (c.C2503G; p.P835A) in the *ITGB4* gene was detected in the patient with UC associated with PSC and SSA. Additionally, *ITGB4* was upregulated in peripheral blood of both UC and PSC patients. It is indicated that mutation of *ITGB4* may affect its expression. The mutation is involved in the pathogenesis of colon cancer [48]. *ITGB4* gene was highly conserved among different species, including humans, *Macaca mulatta*, *Mus musculus*, *Ovis aries*, and *Castor canadensis*. Therefore, *ITGB4* mutation may be considered another candidate molecule in the pathogenesis of this patient.

In addition, a variant, *TP53* (c.86delA; p.N29Tfs^*∗*^15) was identified in the tissue sample of the patient when diagnosed with SSA. Moreover, *TP53* was downregulated in tumor of SSA. It is supposed that mutation of *TP53* may be related to the expression level of *TP53*. Significantly, *TP53* had a potential diagnostic value for SSA, which is considered as a diagnostic biomarker for SSA. *TP53* plays an important role in senescence or apoptosis in cells with damaged genomes [49]. The association between *TP53* mutations and IBD-associated CRC has been reported [50]. A higher frequency of *TP53* mutation is found in UC-CRC compared to sporadic CRC. An increase in the frequency of *TP53* in the bile of PSC patients with the malignancy has been demonstrated. In addition, subsets of serrated adenomas showed *TP53* mutation. Our study indicated that the *TP53* (c.86delA; p.N29Tfs^*∗*^15) mutation may be associated with disease progression of UC associated with PSC and SSA.

In conclusion, mutations in ITGB4 (c.C2503G; p.P835A) and MUC3A (c.C1019T; p.P340L) may predispose patients with UC to PSC and SSA. *MUC3A* is associated with inflammatory bowel disease. These findings may reveal the high degree of genetic heterogeneity in patient with UC associated with PSC and SSA. Additionally, *TP53* (c.86delA; p.N29Tfs^*∗*^15) may be regarded as a risk diagnostic factor for patients developed to SSA. Future work will be performed to improve understanding of this disorder. However, there is a limitation of the present study. First, the sample size is small. Larger peripheral blood and tumor samples from patients with UC associated with PSC and SSA are further needed for analysis. Second, identified mutations are further needed to be verified in colon tumors or precancerous lesions. Third, the in vitro study is further needed to verify the molecular biological changes caused by mutations, such as cell proliferation, dedifferentiation, and tumorigenesis phenotype of intestinal epithelium in cell lines or animal models. Fourth, analysis of gut dysbiosis and differential metabolites is further needed to study in PSC.

## Data Availability

The datasets used and/or analyzed during the current study are available from the corresponding author upon request.

## Supplementary Materials

Supplementary Materials Supplementary Figure 1: distribution of mutated genes (*ITGB4*, *MUC16*, and *TP53*) in SSA and CRC. Supplementary Figure 2: significantly enriched GO terms and KEGG pathways of 73 hotspot mutation genes. The size of the spot indicated counts of mutation genes enriched in GO terms or KEGG pathways, and the color of the spot indicates the FDR/significance level of the enriched pathwasys or GO terms.
