# Supplementary figures and images for: Whole Exome Sequencing Identifies Two Novel Mutations in a Patient with UC Associated with PSC and SSA
Source: Can J Gastroenterol Hepatol. 2021 Sep 10;2021:9936932. doi: 10.1155/2021/9936932 (PMC8449715; doi:10.1155/2021/9936932)

Supplementary

Supplementary Figure 1


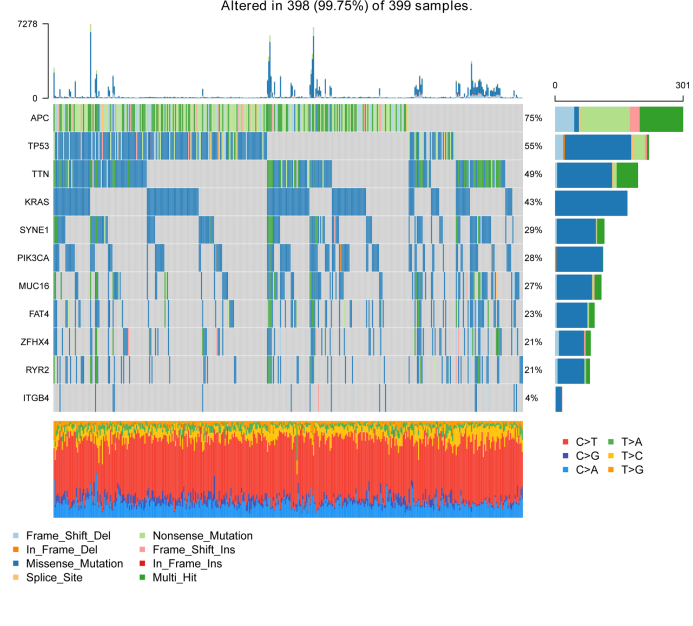


Supplementary Figure 2


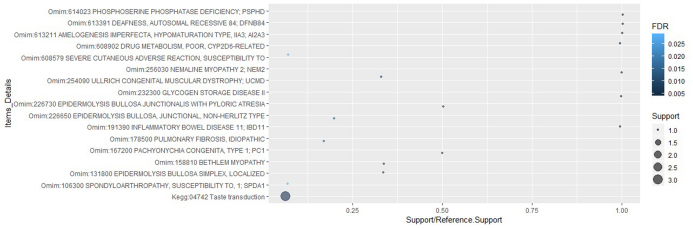

Supplement: Supplementary Materials — Supplementary Figure 1: distribution of mutated genes (ITGB4, MUC16, and TP53) in SSA and CRC. Supplementary Figure 2: significantly enriched GO terms and KEGG pathways of 73 hotspot mutation genes. The size of the spot indicated counts of mutation genes enriched in GO terms or KEGG pathways, and the color of the spot indicates the FDR/significance level of the enriched pathwasys or GO terms. [file 9936932.f1.docx]
